# Early and late assessment of renal allograft dysfunction using intravoxel incoherent motion (IVIM) and diffusion-weighted imaging (DWI): a prospective study

**DOI:** 10.1007/s00261-024-04470-x

**Published:** 2024-07-08

**Authors:** Mostafa El-Ksas, Dina EL-Metwally, Dalia Fahmy, Haytham Shebel

**Affiliations:** 1 Radiology Department, Urology and Nephrology Center, Mansoura University, El Gomhoureya St, Mansoura, Egypt; 2 Radiology Department, Mansoura University, Mansoura, Egypt

**Keywords:** Renal allograft, Intravoxel incoherent motion (IVIM), Estimated glomerular filtration rate (eGFR), Renal function, Impaired renal allograft function, Renal biopsy

## Abstract

**Purpose:**

To evaluate the ability of the Intravoxel Incoherent Motion (IVIM) and monoexponentially ADC in renal allograft function in the early and late phases of transplantation, and to predict their effectiveness in discrimination of the graft pathology.

**Methods:**

This is a prospective study included participants scanned with quantitative diffusion and perfusion sequences on a 3-T MR scanner (Philips, Ingenia); the ADC and IVIM parameters; were calculated. Correlations and regression analysis with the eGFR, transplantation periods, and pathology were assessed.

**Results:**

This study included 105 renal allograft recipients (85 males, and 20 females with mean age = 32.4 ± 11.9 years and age range = 22–61 years). There was a significant positive correlation between the whole parameters of the ADC and IVIM with eGFR however, the cortical parameters showed higher significant correlation coefficients (*p* < 0.001). Regression analysis revealed the most significant model can predict eGFR groups included cortical pseudo diffusion (D*) and cortical ADC (*p* < 0.001). In graft dysfunction eGFR was 61.5 ml/min and normal graft was 64 ml/min. This model demonstrates a high performance of an AUC 96% [0.93–0.97]. In the late transplantation, there is a higher correlation with D* compared to ADC, *p*-values = 0.001.

**Conclusion:**

IVIM and ADC Values are significant biomarkers for renal allograft function assessment, cortical ADC, and D* had the highest performance even in situations with mild impairment that is not affect the eGFR yet as cases of proteinuria with normal eGFR. Furthermore, D* is superior to ADC in the late assessment of the renal transplant.

**Graphical abstract:**

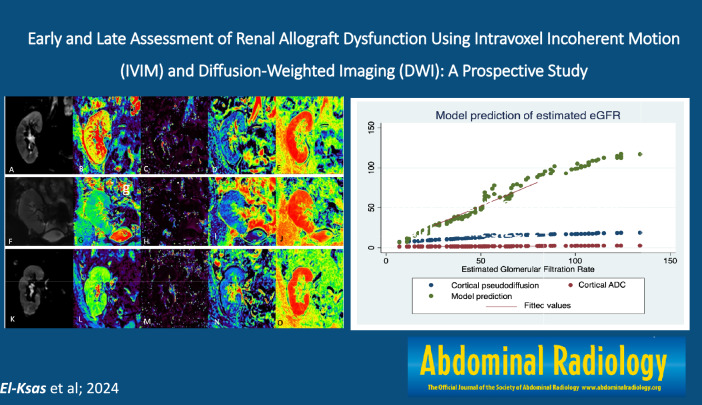

**Supplementary Information:**

The online version contains supplementary material available at 10.1007/s00261-024-04470-x.

## Introduction

The ideal alternative for end-stage renal disease is renal allograft transplantation since it boosts long-term survival rates and improves patient quality of life [1, 2]. One frequent post-transplant consequence is renal allograft impairment [1, 3, 4], that includes acute rejection (AR), acute tubular necrosis (ATN), cyclosporine toxicity, delayed graft function (DGF), pyelonephritis (PN), and chronic kidney injury (CKI). The clinical symptoms of various etiologies may be identical, yet the treatments are very dissimilar. The decision management to guarantee a positive outcome for renal allograft transplantation needs a crucial and close monitor that promptly addresses renal allograft impairment in the early and late stages.

Although ultrasonography (US) is a useful tool for general evaluation of renal allograft function, especially by using different Doppler patterns, some renal allograft problems can be missed by US parameters, which also depend on the operators [5–7]. The best evidence for acute renal allograft damage can be assessed by percutaneous renal needle core biopsy, although it is invasive has potential for different minor and major complications [8, 9]. Another imaging modality is renal scintigraphy, which measures glomerular filtration rate (GFR), is thought to be the best indicator of renal allograft function. However, its use is declining as renal imaging agents have to some extent a high radiation exposure and are inaccurate for differentiating different underlying patterns of graft dysfunction. The current cross-sectional imaging, as computed tomography and anatomical MRI sequences can diagnose many surgical post-transplant complications such as urinary leaks, peri transplant collections, obstruction, and vascular complications; however, it cannot determine the degree of the graft function impairment caused by the aforementioned or other common immunological complications. The development of functional MRI sequences in recent decades has the potential to produce a promising imaging biomarker that can non-invasively investigate oncological status in different parts of the body and assess the renal function in a variety of urinary tract disorders as well as renal transplant dysfunction. [10–17, 22].

Diffusion-weighted imaging (DWI) is a promising non-invasive technique for evaluating renal function and different urinary tract disorders [10, 11] including renal allograft performance, due to its high sensitivity to changes in the free water movement within the biological tissue’s microstructure [12–17]. Signals of DWI were quantified by the apparent diffusion coefficient (ADC) with no need for contrast injection. Previous research demonstrated that compromised renal allografts have a much lower apparent diffusion coefficient (ADC) value than normal renal allografts [12, 13, 16, 18]. Flow-induced pseudo-diffusion is particularly noticeable in the renal tissue as a consequence of the significant microscopic flow caused by water reabsorption, blood filtration, and urine generation. Multiple b-values DWI experiments using intravoxel incoherent motion (IVIM), which uses the biexponential model for MR signal intensity decay, can separate between true diffusion and pseudo-diffusion [19, 20]. The effectiveness of IVIM in the assessment of various renal disorders has been proven in a number of studies [21–27]. Few studies have combined both ADC and IVIM to evaluate the performance of both cortical and medullary values for identifying renal allograft impairment. Furthermore, the majority of research focused on evaluating graft function in the initial post-transplant phase rather than in the later phase [28, 29]. Therefore, our primary objective is to investigate the correlation pattern and its strength between IVIM and monoexponentially ADC with the graft function in the early and late transplantation periods using eGFR as a standard biomarker. The secondary objective is to explore the ability of both markers to differentiate between different pattern of the graft dysfunction.

## Material and methods

### Study population

The present prospective study was permitted by the Institutional Review Board, and informed written consent was given by each participant. The data of the patients remains anonymized. Between October 2021 and January 2023, renal allograft recipients were referred to the magnetic resonance imaging unit, from the dialysis and kidney transplantation unit. The Inclusion criteria included 57 patients with different transplant periods and abnormal graft function to assess the capability of the MRI predictors in the assessment of transplant function within early and late stages. Less than 1 year (represents early transplant cohort), 1 to 5 years (for moderate time cohort), and more than 5 years (as late or older time cohort) with elevated serum creatinine and an abnormal estimated glomerular filtration rate (eGFR). All cases with abnormal lab function were scheduled for an allograft biopsy procedure. Another matched group of 48 cases regarding the duration of the transplant period, age, and sex with normal allograft function was selected as a control group, and a flow chart for inclusion criteria was included as a supplement 1. Prior to an MR examination, ultrasonography was routinely carried out; renal allograft recipient patients with vascular difficulties, collection of fluids, urologic issues, malignancies, and bilateral renal transplantation were disqualified.

Serum creatinine levels of all cases were measured on the same day of the MRI study and were used to compute the estimated glomerular filtration rate (eGFR) consistent with the CKD Epidemiology Collaboration (CKD-EPI) Creatinine Equation. Cases were divided into two groups according to their eGFR. cases with good renal allograft function (eGFR ≥ 60 mL/min/1.73m^2^, and cases with impaired renal allograft function (eGFR < 60 mL/min/1.73m^2^.The patients were withdrawn from water intake in addition to intravenous fluid intake for at least 2 h before the MRI study.

### MRI study

All MRI studies were done on a 3 Tesla MRI scanner (Philips, Ingenia) with a phased-array body coil in the supine position. For anatomic characterization, coronal T1-weighted and axial T2-weighted images were routinely acquired. IVIM imaging was performed in the oblique sagittal plane without breath-hold using a single-shot diffusion-weighted echo-planar imaging (EPI) sequence (TE/TR = 72.4/1800 ms; 5 slices; slice thickness = 5 mm; FOV = 230 × 230mm^2^; matrix = 128 × 128; 14 b values at 0, 5, 10, 20, 30, 40, 50, 60, 100, 150, 200, 300, 400, 500 s/mm^2^; parallel imaging generalized auto calibrating partially parallel acquisition [GRAPPA] factor *r* = 2 on three gradient directions). The respiratory-triggered technique was not mandatory, as the renal allografts were located in the iliac fossa either right or left, and the respiratory motion artifacts were not insignificant. The average acquisition time was around 5 min.

### Image analysis

Following image acquisition, all images were stored in DICOM format, then transferred to a digital workstation (Intellispace portal Workspace 6.0.1 Philips Medical Systems, Netherlands B.V) supplied by the vendor, for processing. Diffusion parametric maps were measured by an IVIM processing tool in which all three IVIM parameters, diffusion coefficient (D), pseudo-diffusion coefficient (D*), and perfusion fraction (*f*), were measured by a full biexponential fitting of the MR signal intensity decay consistent with the equation: S(b)/S(0) = (1_f) × exp (– b × D) + f x exp (− b × D*); wherein S(b) denotes the signal intensity whereas the diffusion sensitization is present and S(0) denotes the signal intensity whereas the diffusion sensitization is absent. D (10^−3^mm^2^/s) denotes predominantly pure molecular diffusion, as well as D* (10^−3^mm^2^/s) denotes the pseudo-diffusion coefficient dominated by the much faster microcirculation or perfusion; and *f* (%) denotes the perfusion fraction (i.e., the contribution of microcirculation of blood and movement in predefined structures, such as tubular flow to the signal decay) [25].

The ADC values [cortical ADC (CADC), medullary (MADC), and total ADC (TADC)] were measured by a mono-exponential fitting of the MR signal intensity decay consistent with the equation: S(b)/S(0) = exp (– b × ADC) [21].For ADC, a large ROI that covers the entire cortex and three (ROIs), about 20–30 pixels were drawn in the upper, middle, and lower portions of the medulla (m) in each slice on the images of b = 0 s/mm^2^, and then copied to the matching ADC, D, D*, and *f* maps. The whole images were examined by two independent radiologists [Shebel H and El-Kasas M] who have 25- and 10- year experience in the pelviabdominal MRI and there was a consensus regarding the position of the ROI, and they were blinded to the eGFR or biopsy results of all cases.

### Statistical analysis

STATA/IC version16.1 statistical software Stata-Corp LLC, USA, was used for the statistical analysis. The paired t-test was used to compare variations in each group's cortex and medulla. Independent sample t-tests were used to compare differences between the two groups. In renal allografts, correlations between eGFR and the IVIM parameters were evaluated using Spearman correlation analysis. The diagnostic usefulness of using IVIM and ADC parameters to distinguish between renal allografts with impaired function and those with good function was evaluated using a receiver operating characteristic (ROC) curve analysis. Binary stepwise logistic regression analysis was used for the prediction of the independent variables of impaired renal allograft function. Significant predictors in the univariate analysis were entered into the regression model. Adjusted odds ratios and their 95% confidence intervals are calculated. Results with *p* values < 0.05 were considered statistically significant.

## Results

This study included a total of 105 renal allograft recipient patients (85 males and 20 females, with a mean age of 32.4 ± 11.9 years and an age range of 22–61 years). Our results showed cases with normal eGFR (*n* = 48) in 38 men and 10 women, with a mean age of 32.4 + 12.4 years and a mean eGFR of 89.85 + 20.99 mL/min/1.73 m^2^. While cases with abnormal eGFR (*n* = 57, 47 men and 10 women, mean age = 32.4 + 11.6 years, and mean eGFR = 37.02 + 13.57 mL/min/1.73 m^2^). Regarding the transplantation period, for less than 1 year, there were 33 cases for the cohort study and 29 cases for the control group; for a 1-to-5-year cohort, there were 10 and 11 cases for the control and study groups, respectively; and for more than 5 years, there were 9 and 13 cases for the control and study groups. All of the 57 recipient cases with impaired renal allograft function received a percutaneous renal needle core biopsy that showed AR in 22 cases, ATN in 12 cases, and CKI in 23 cases. Both IVIM and CADC parameters for all groups are provided in Table 1.

**Table 1:** ADC and IVIM parameters of the renal cortex and renal medulla in normal and impaired renal allograft function

	Good function (*N* = 48)	Impaired function (*N* = 57)	*P*-value
CADC	2.29 ± 0.28	1.71 ± 0.13	*p* ≤ 0.001*
MADC	2.22 ± 0.28	1.69 ± 0.13	*p* ≤ 0.001*
CD	1.65 ± 0.05	1.51 ± 0.06	*p* ≤ 0.001*
MD	1.59 ± 0.06	1.51 ± 0.06	*p* ≤ 0.001*
CPD	16.64 ± 1.2	11.56 ± 2.2	*p* ≤ 0.001*
MPD	15.73 ± 1.1	11.27 ± 2.1	*p* ≤ 0.001*
CF	0.34 ± 0.04	0.23 ± 0.02	*p* ≤ 0.001*
MF	0.32 ± 0.04	0.23 ± 0.03	*p* ≤ 0.001*

Data expressed as mean ± *SD*

*CADC* cortical ADC, MADC, medullary ADC, *CD* cortical diffusion coefficient, *MD* medullary diffusion coefficient, CPD cortical pseudo diffusion, *MPD* medullary pseudo diffusion, *CF* cortical perfusion fraction, *MF* Medullary perfusion fraction

*Statistically significant (*p* < 0.05)

### Assessment of the graft function predictors

#### Analysis of ADC parameters

**Fig. 1:**
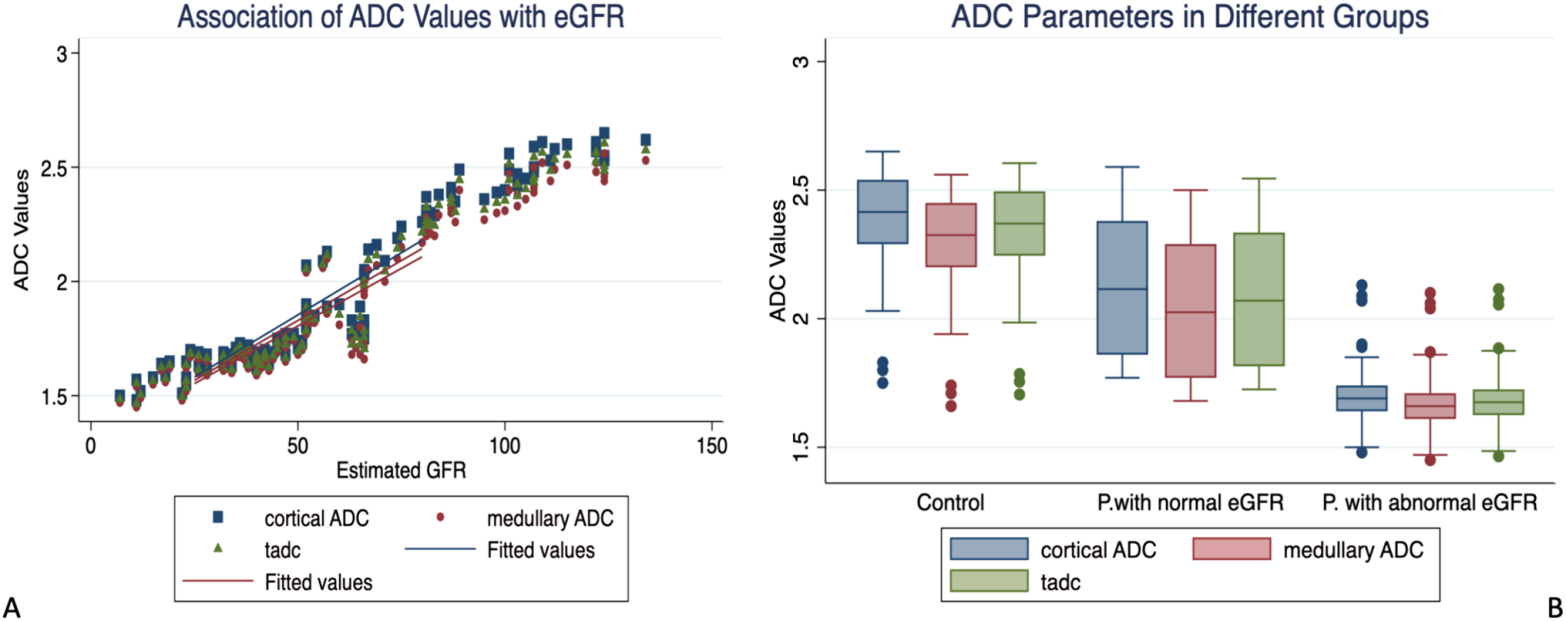
**A** Scatter plot graph shows positive correlation between cortical, medullary, and total ADC with estimated eGFR.** B** box and plot graph show ADCs are higher in control groups compared with patients with normal eGFR but with proteinuria. While patients with abnormal eGFR shows least ADCs values. *ADC*: Attenuation Diffusion Coefficient, *Cortical ADC* Cortical Attenuation Diffusion Coefficient, *Medullary ADC* Medullary Attenuation Diffusion Coefficient, *tadc* Total Attenuation Diffusion Coefficient, *eGFR* estimated glomerular filtration rate

There was a significant difference in the ADC values [CADC, MADC, and TADC] within the three groups [control, normal eGFR without proteinuria, and abnormal eGFR with proteinuria] (*p* < 0.001). In all groups, CADC had a higher value than MADC and TADC. Moreover, there is a strong positive association between the values of all ADCs and eGFR. There is a strong positive correlation between ADC and eGFR. Interestingly, the ADC values differed between the control group and patients with normal eGFR but with proteinuria Fig. 1**.** Regression analysis included the three ADC values that were applied to find out the margin’s prediction of eGFR in the different above mentioned groups (Supplement 2). Further secondary analysis to establish the best combination model of ADCs revealed CADC is the single significant parameter among other ADCs whose values can predict and highly correlated with eGFR (*p*-value = 0.02) in different renal groups Table 2**.** Therefore, CADC will step forward in the next phase of analysis.

**Table 2:** Cortical ADC predicted eGFR in different groups

	Margin	STD	Z	P > Z	[95% confidence interval]
Renal_function_groups					
Control	69.5102	3.746677	18.55	0.001	62.16685–76.85356
PATIENTS with normAL eGFR but with proteinuria	65.36735	3.708652	17.63	0.001	58.09852–72.63617
Patients with abnormal eGFR	57.36735	2.657579	21.59	0.001	52.15859–62.57611

Table describes the predicted eGFR margin for each group with 95% confidence interval

*STD* Standard deviation

#### Analysis of IVIM parameters

**Fig. 2:**
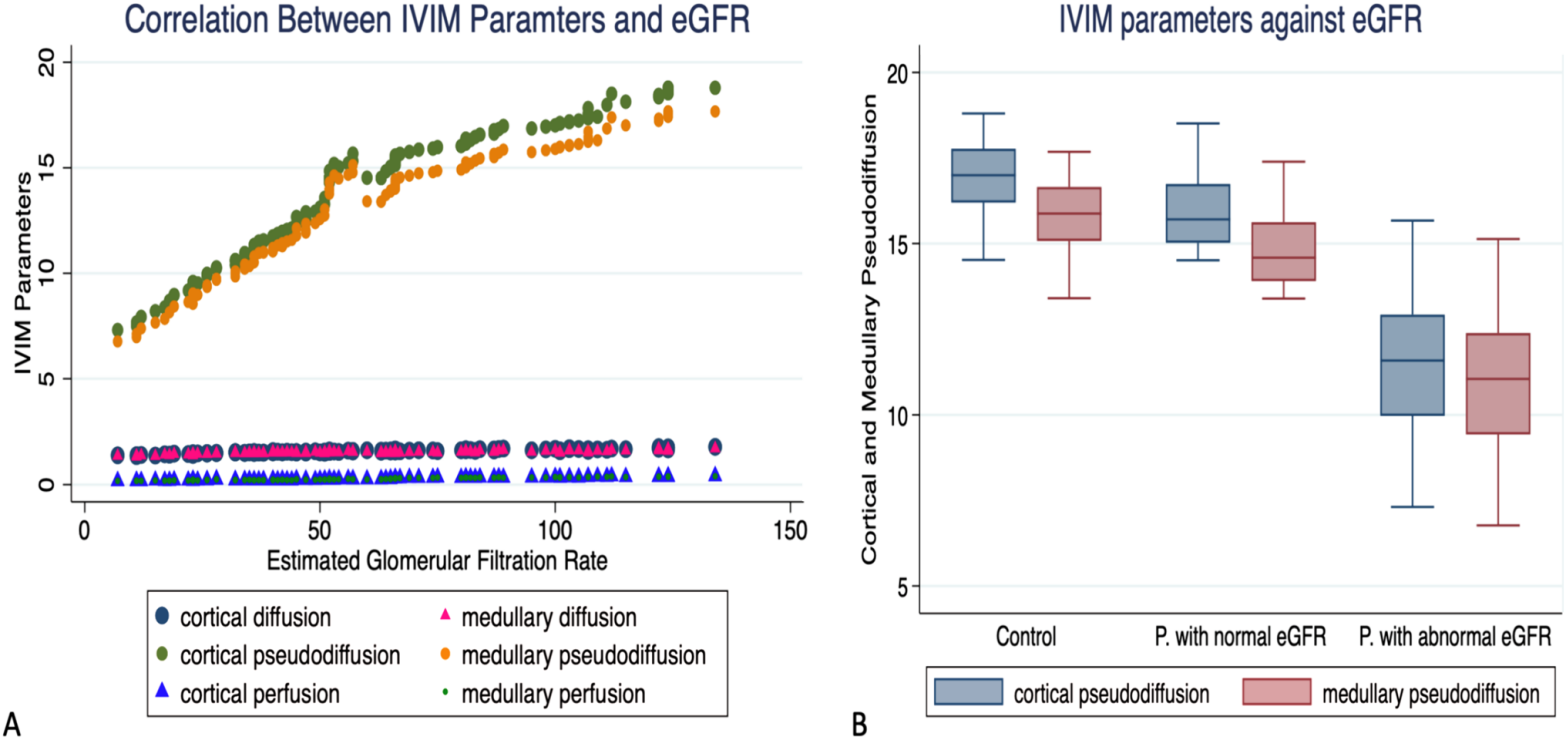
**A** A scatterplot shows the correlation between IVIM parameters with estimated eGFR, cortical and medullary pseudo diffusion show the highest positive correlation. **B** box and plot graph show both cortical and medullary pseudo diffusion are higher in control groups compared with patients with normal eGFR but with proteinuria. While patients with abnormal eGFR shows least values. *IVIM* Intra-voxel incoherent Motion, *eGFR* estimated glomerular filtration rate

Regarding IVIIM parameters, there was a significant positive correlation between the whole parameters and eGFR; however, the cortical parameters showed higher significant correlation coefficients. Cortical and medullary pseudo-diffusion were the most predictors highly correlated with eGFR (*p* =  < 0.001). Furthermore, there is a significant difference of the IVIM parameters within the different groups (*p* = 0.003) Fig. 2. Cortical pseudo-diffusion was the most significant predictor (*p* =  < 0.001) for the eGFR in different renal groups that showed similar interesting changes in ADC values that combined normal eGFR but with proteinuria. Supplement 3. Therefore, it will move forward in the next phase of analysis Table 3.

**Table 3:** Cortical pseudo-diffusion [CPD] predicted eGFR in different groups

	Margin	STD	Z	P > Z	[95% confidence interval]
Renal_function_groups					
Control	75.93427	2.77726	27.34	0.001	70.49094–81.3776
Patients with normal eGFR but with proteinuria	57.1761	3.125614	18.29	0.001	51.05001–63.30219
Patients with abnormal eGFR	55.31989	2.083335	26.55	0.001	51.23663–59.40315

Table describes the predicted eGFR margin for IVIM [cortical pseudo diffusion, CPD] within each group with 95% confidence interval

*STD* Standard deviation

#### Combined model prediction

**Fig. 3:**
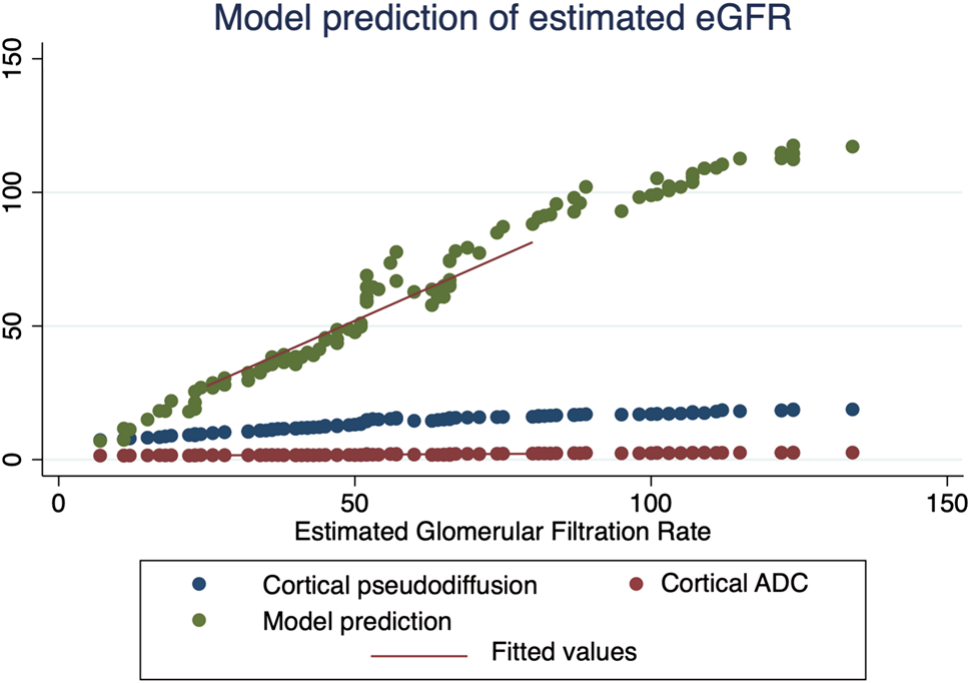
The scatterplot graph shows the combined model has a higher prediction and correlation with eGFR compared with cortical pseudo-diffusion (CD*) and cortical ADC (CADC) separately in the different groups. *ADC* Attenuation Diffusion Coefficient, *CADC* Cortical Attenuation Diffusion Coefficient, CD* Cortical pseudo diffusion

Stepwise regression analysis revealed the most significant variables that can predict graft status based on eGFR were D* and CADC (*p* 0.001), with a higher correlation with D*. The predicted margin of graft dysfunction was 61.5 ml/min. and that of normal graft function was 64 ml/min. The model that contains these two parameters outperforms each single parameter and achieved a high performance up to an area under the curve equals 96% [0.93–0.97] CI Fig. 3. This model represents the most valuable predictors that can be used in the follow up and management strategies for such patients.

#### Correlation with the transplant duration

**Fig. 4:**
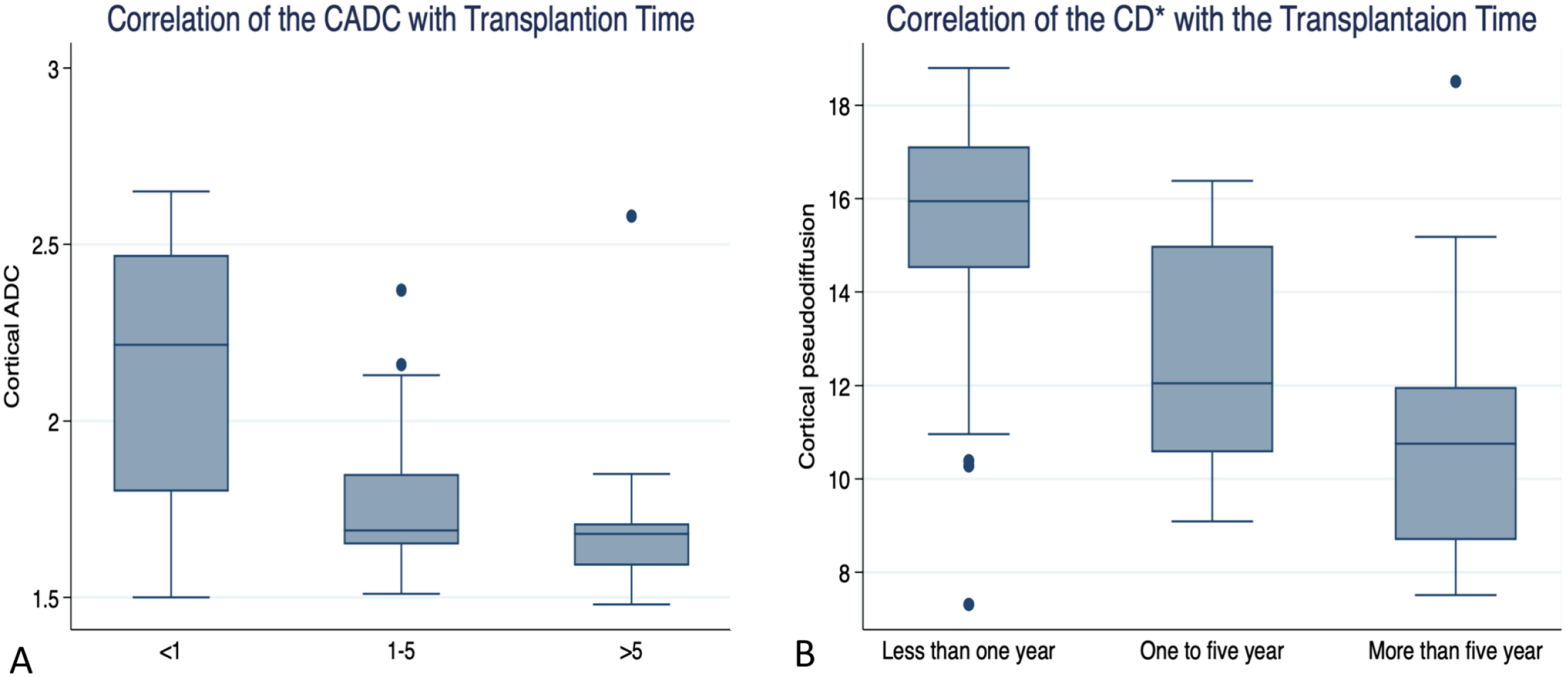
Box and plot shows how both cortical ADC (CADC) and cortical pseudo-diffusion (CD*) values change with eGFR with different duration of the renal allograft transplantation. A There is a drop of ADC value after one year and then become constant. B cortical pseudo-diffusion (CD*) shows continuous decline change for its value with the prolonged duration of the graft time and observed changes from one year to more than five years. *ADC* Attenuation Diffusion Coefficient, CD* Cortical pseudo diffusion

The correlation of CADC and D* with allograft duration revealed that D* is a sensitive parameter that is negatively associated with the time of the transplanted kidneys. Using the Kruskal–Wallis test, there is a significant difference in the decrease in D* values between transplant durations of < 1 year, 1–5 years, and > 5 years (*p*-value = 0.001), whereas CADC showed a decrease after 1 year but remained stable over 5 years. Figure 4 and Table 4 this means that D* is more sensitive and reliable compared to ADC values when following patients with extended transplant times.

**Table 4:** Graft duration analysis between CADC and CPD

	Variable	Cases	Mean	Median	Min	Max
Transplant duration = < 1 year	CADC	62	2.1	2.2	1.5	2.65
	CPD	62	15.4	15.8	7.3	18.8
Transplant duration = 1–5 years	CADC	21	1.7	1.7	1.5	2.4
	CPD	21	12.5	12.2	9.1	16.4
Transplant duration = > 5 years	CADC	22	1.7	1.7	1.48	2.58
	CPD	22	10.8	10.8	7.51	18.51

Table describes the point estimate parameters [ mean, median, minimum, and maximum] of both cortical ADC and cortical pseudo diffusion, CPD in comparison to different transplant periods

### Model assessment in the prediction of the graft pathology

**Fig. 5:**
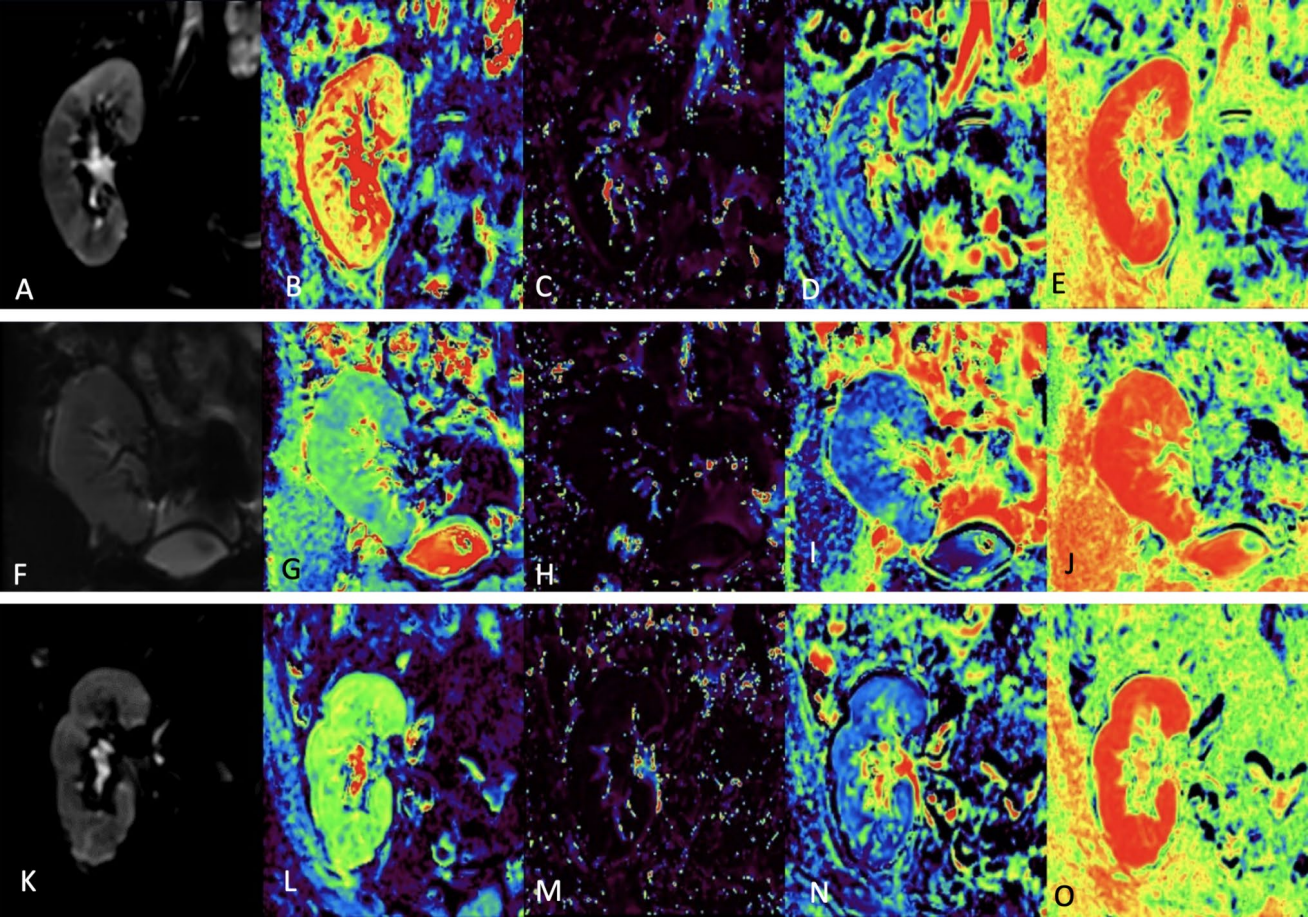
**A**–**E** was for a 21-year-old male with renal allograft transplantation 9 months ago with good renal allograft function; his scr level was 1.1 mg/dL with eGFR about 98 mL/min/1.73m^2^. IVIM-DWI MR parameter maps **A** b0 image, **B** D map, **C**D* map, **D** f map, and **E** Goodness of Fit. **F**–**J** was for a 25-year-old male with renal allograft transplantation 3 years ago presented with severe renal allograft impairment; his sCr level was 7.6 mg/dL from basal sCr level about 1.1 mg/dL with eGFR about 9 mL/min/1.73m^2^. He underwent renal allograft biopsy and was diagnosed as renal allograft rejection. IVIM-DWI MR parameter maps **F** b0 image, **G** D map, **H** D* map, **I** f map, and **J** Goodness of Fit. **K**–**O** was for a 53-year-old male with renal allograft transplantation 6 years ago presented with mild renal allograft impairment; his sCr level was 1.6 mg/dL from basal sCr level about 0.9 mg/dL with eGFR about 51 mL/min/1.73m^2^. He underwent renal allograft biopsy and was diagnosed as tubular necrosis on top of chronic renal allograft fibrosis. IVIM-DWI MR parameter maps **K** b0 image, **L** D map, **M** D* map, **N** f map, and **O** Goodness of Fit. *D* Diffusion coefficient, D* Pseudo-diffusion coefficient, *F* Perfusion fraction

There was a significant difference between CADC and CD* in differentiating rejection from non-rejection cases (*p*-values = 0.003, 0.001) respectively. The combined model showed high significance performance with an AUC 80% [0.67–0.85] CI. While CADC revealed a negative correlation, CD* showed a positive one with rejection cases. Furthermore, the same model fails to differentiate between ATN and non ATN cases or CKD and non-CKD cases. When using D* and CADC as predictors of eGFR for these groups, acute rejection and CKD had the two lowest values, while ATN and control groups showed the highest values (Supplement 4) with significant *p*-values (0.01). The IVIM perfusion map is shown in Fig. 5.

## Discussion

The present study revealed that IVIM analyses including DWI provided multiple meaningful results. ADC values showed a substantial positive correlation with eGFR, with cortical values having larger coefficients than medullary values. This correlation was particularly sensitive when comparing a group with normal eGFR but with proteinuria and a completely normal group, Fig. 1, Table 1, and Supplement 2. This suggest that ADC levels were linked to and sensitive to transplant function even in patients with normal eGFR but with proteinuria. Our thorough analysis demonstrated that CADC was the best predictor of all ADC values and can be used as a standalone marker for graft function assessment when conventional DWI is used. These findings are consistent with earlier research that supports the same conclusion, which can be related to the increased blood perfusion of the cortex and the restricted diffusion of the medulla [17, 30], resulting in a strong positive association with eGFR values, either increasing or decreasing [27, 31–34]. However, the ability of the monoexponentially models of ADC calculations to distinguish between diffusion and perfusion effects is restricted, which might result in bias when different b-values are employed across studies [18]. Fitting signal attenuation in IVIM using a biexponential model is an efficient way for improved mathematical fitting and a more accurate description of DWI signals in renal allografts [25].

The cortical and medullary markers of IVIM [D, D*, and f] were substantially correlated with graft function in our data, and cortical values were much more correlated than medulla in renal allografts various groups’ function. Further analysis of the IVIM parameters demonstrated pseudo-diffusion was the most significant parameter can be used for the accurate assessment of the graft function when using the model prediction analysis Fig. 2, Table 2, and Supplement 3. This can be explained by the fact the impairment of a renal allograft can be caused by a variety of factors. One of the important factors includes endothelial ischemia, which can cause cell swelling and damage, reduced blood flow, reperfusion, which primarily represents ischemia reperfusion injury, and interstitial inflammation. [3, 35]. Also, the higher cortical correlation parameters could be explained by the fact that the higher blood perfusion of the renal cortex than the renal medulla makes the cortex experience a more pronounced reduction in blood circulation than the medulla. This outcome is in accordance with earlier studies [36, 37]. However, in these studies, the main concern of the authors was to predict the single marker which can predict graft function. In the present study, when comparing impaired and normal function, the diagnostic value of using all cortical parameters simultaneously is clearly greater than using one parameter alone or all medullary parameters together. This means that the ability to diagnose renal allograft deterioration using combined cortical parameters has increased. The secondary analysis and model building strategy revealed that the combination of CADC and D* in one model can predict graft function with excellent performance and can effectively distinguish between normal and abnormal renal functional allografts with an AUC 96% [0.93–0.97] CI. This model outperformed any single predictor for IVIM markers or standalone ADC Fig. 3

### Correlation with the transplant duration

One of the interesting results presented in the current study is the high negative correlation of IVIM parameters, especially [D* and CPF] with transplant duration compared with CADC. In our study, IVIM showed a significant reduction after 1 year and a continuous reduction after 5 years of transplantation. On the other hand, CADC showed only a reduction in its value after 1 year, which persisted with the same value even after 5 years of duration Fig. 4 and Table 4. Many studies in accordance with our results, despite their small sample size, as Sułkowska et al. [38] studied the value of ADC in a small sample size in the early transplant period [5–19 days] and documented the reduction of both ADC and fraction perfusion in early graft dysfunction. Another small sample size study conducted by Chang et al. [39] concluded that diffusion and perfusion indexes correlated significantly with serum creatinine concentrations in the early transplant period. On the other hand, few studies have investigated ADC and IVIM over a lengthy period of time after transplantation, as this study did. Eisenberger et al. [40] investigated both metrics in CKD patients with native kidneys and observed that while both markers were positively correlated with eGFR, CD* had a more substantial correlation with the degree of fibrosis that developed over time.

### Prediction of the graft **pathology**

With an AUC of [80%] [0.67–0.85] CI, our model can predict rejection from non-rejection situations. This is consistent with previous findings that employed only total ADC without separation and concluded that ADC can predict cases of acute rejection from ATN patients but cannot identify ATN from other cases even in the normal group [41].

These findings suggest the potential for a successful novel imaging biomarker that may be incorporated and utilized in routine clinical practice to aid in the monitoring of renal allograft patients with varying transplantation durations. The ADC can examine patients for potential abnormalities before eGFR alterations are observed, particularly in the first year. IVIM, on the other hand, demonstrates a greater correlation coefficient in the follow-up period after one and even 5 years post transplantation. These potential imaging indicators prompt us to urge additional large-scale multicenter investigations to validate their accuracy. Longitudinal studies are another type of study design that is highly recommended for following up on these patients and recording the degree and pattern of the correlation with different episodes of graft survival.

## Limitations

Although our study has strengths, there are limitations that should be acknowledged. First, it is a single center study that might affect the generalizability of the results; however, the findings encourage conducting a larger multicenter study with the same protocols to enhance the reproducibility and validate the clinical relevance of the findings. Second, there is no follow up for the patients who exhibit deteriorated graft function after improvement; however, these initial results are important to identify which useful parameters could be utilized on a larger scale in the future study. Third, the relatively small number of patients in the subgroups, however, doesn’t violate the sample size calculation which preceded the study design to provide 80% power. Furthermore, the sample size employed was larger than many of the publications cited in the literature. Fourth, the study may be subject to selection bias because participants were referred from the same institution clinic throughout a certain time period.

## Conclusions

This study concluded that MRI imaging biomarkers have the potential to be used in the follow-up of renal allograft patients, either in the early or late phases of transplantation. IVIM has superior performance in the early and late phases; however, ADC can be utilized as a good alternative option only in the first year. Also, these markers demonstrated its potential ability to differentiate rejection and non-rejection cases. However, it failed to demonstrate satisfactory results in differentiating other causes of graft dysfunction other than rejection.cases. Furthermore, both sequences are highly correlated with eGFR as a standard biomarker. One of the interesting outcomes of this study is that both markers can predict early graft dysfunction even with normal eGFR, as the eGFR margin in the cohort with proteinuria and normal eGFR is less than the margin of the control cohort but higher than the cohort with proteinuria and abnormal eGFR. These findings demonstrate the potential of utilizing these biomarkers in the routine investigation and follow-up of such patients, help in decision management, and should direct future research to be implemented on a large scale, including multicenter and longitudinal studies.

## Supplementary Information

Below is the link to the electronic supplementary material.

Supplementary file1 (DOCX 169 KB)

Supplementary file2 (DOCX 64 KB)

Supplementary file3 (DOCX 71 KB)

Supplementary file4 (DOCX 177 KB)

## Data Availability

The datasets used and/or analyzed during the current study are available from the corresponding author on a reasonable request.

## Abbreviations

AR: Acute rejection
ATN: Acute tubular necrosis
DGF: Delayed graft function
PN: Pyelonephritis
CKI: Chronic kidney injury
GFR: Glomerular filtration rate
DWI: Diffusion-weighted imaging
ADC: Apparent diffusion coefficient
IVIM: Intravoxel incoherent motion
D: Diffusion coefficient
D*: Pseudo-diffusion coefficient
f: Perfusion fraction
