# Supplementary figures and images for: Early and late assessment of renal allograft dysfunction using intravoxel incoherent motion (IVIM) and diffusion-weighted imaging (DWI): a prospective study
Source: Abdom Radiol (NY). 2024 Jul 8;49(11):3902–12. doi: 10.1007/s00261-024-04470-x (PMC11519223; doi:10.1007/s00261-024-04470-x)

**Flow chart for inclusion criteria**


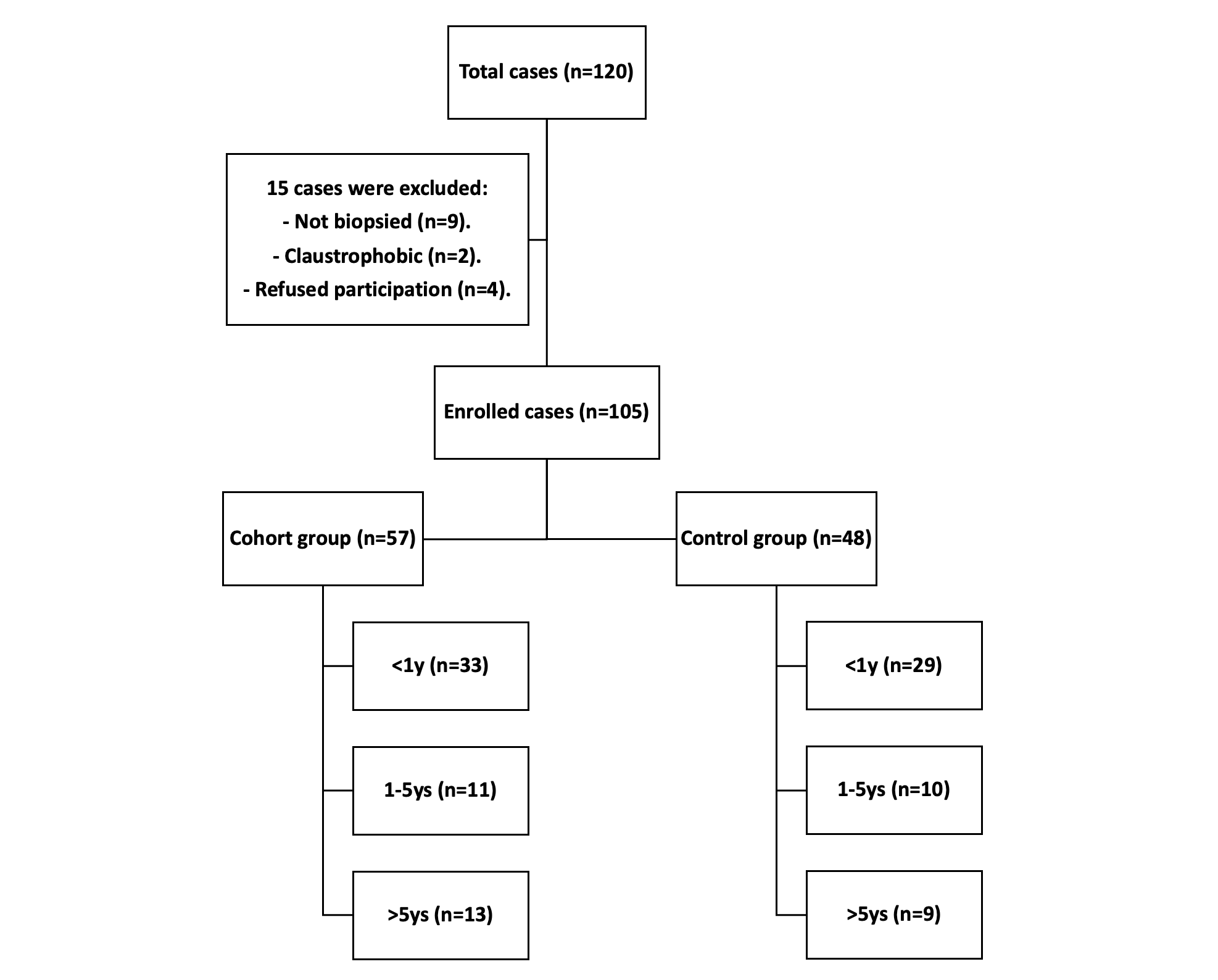

Supplement: Supplementary file 1 — Supplementary file1 (DOCX 169 KB) [file 261_2024_4470_MOESM1_ESM.docx]
