# Supplementary material for: Early and late assessment of renal allograft dysfunction using intravoxel incoherent motion (IVIM) and diffusion-weighted imaging (DWI): a prospective study
Source: Abdom Radiol (NY). 2024 Jul 8;49(11):3902–12. doi: 10.1007/s00261-024-04470-x (PMC11519223; doi:10.1007/s00261-024-04470-x)
Supplement: Supplementary file 2 — Supplementary file2 (DOCX 64 KB) [file 261_2024_4470_MOESM2_ESM.docx]

**Supplement 2**

Fig1: Regression analysis of ADC with the eGFR in different cohort of the studies. The control group shows the highest eGFR followed by cohort with normal eGFR but with proteinuria and the lowest eGFR predicted values in the cohort that showed abnormal eGFR and abnormal proteinuria.
