# Supplementary material for: Early and late assessment of renal allograft dysfunction using intravoxel incoherent motion (IVIM) and diffusion-weighted imaging (DWI): a prospective study
Source: Abdom Radiol (NY). 2024 Jul 8;49(11):3902–12. doi: 10.1007/s00261-024-04470-x (PMC11519223; doi:10.1007/s00261-024-04470-x)
Supplement: Supplementary file 4 — Supplementary file4 (DOCX 177 KB) [file 261_2024_4470_MOESM4_ESM.docx]

**Supplement 4**

A

B


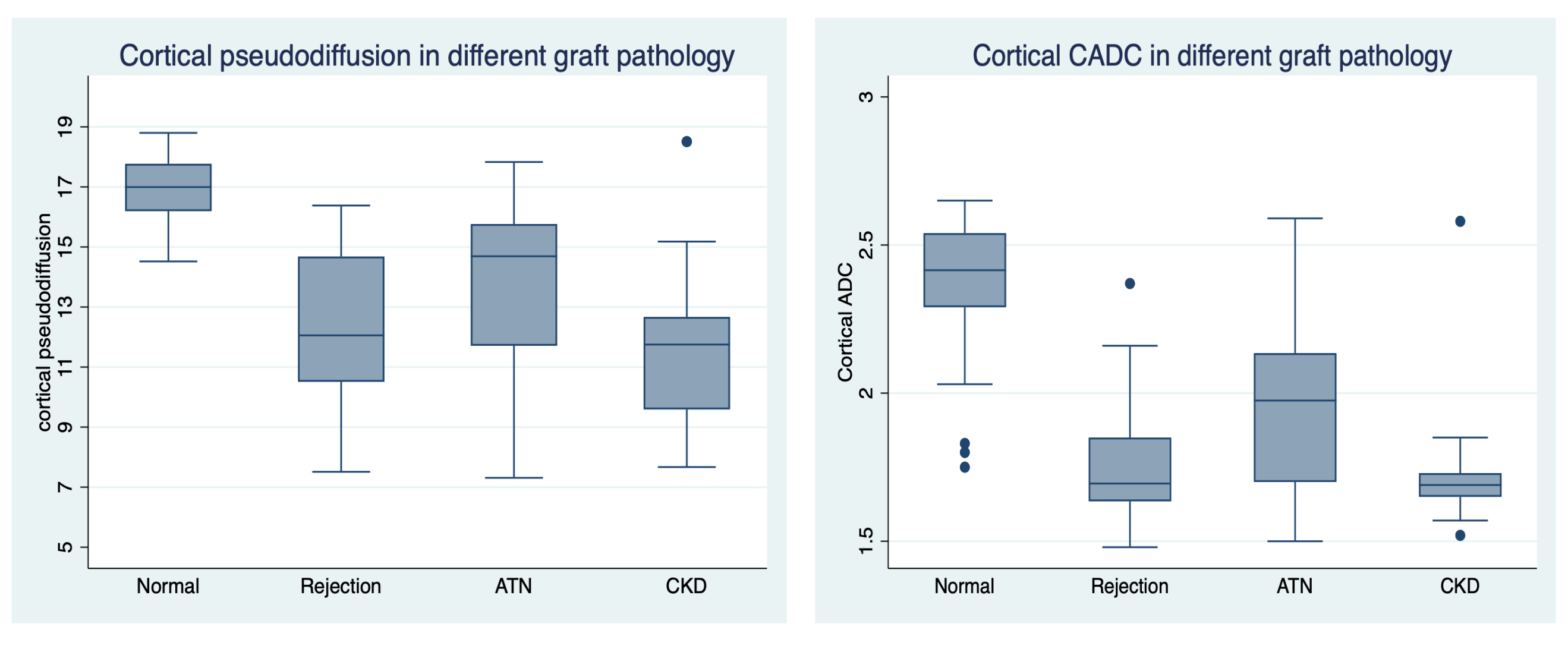
**Fig:** Box and Plot Graph showed: Both CADC and cortical pseudo-diffusion (A,B) show rejection and chronic kidney disease (CKD) have lowest values. While normal group and acute tubular necrosis ATN exhibits highest values with both parameters. There was a significant difference between CD*and CADC in differentiating rejection from non-rejection cases (p-values = 0.001, 0.003) respectively.
